# Differentiating Immune-Related Adrenal Insufficiency From Low Cardiac Output Syndrome: A Case Report

**DOI:** 10.7759/cureus.31349

**Published:** 2022-11-10

**Authors:** Junya Tanabe, Nobuhide Watanabe, Mayuna Ito, Keizo Kanasaki, Kazuaki Tanabe

**Affiliations:** 1 Cardiology, Shimane University Faculty of Medicine, Izumo, JPN; 2 Endocrinology and Metabolism, Shimane University Faculty of Medicine, Izumo, JPN

**Keywords:** squamous cell lung cancer, adrenocortical insufficiency, heart failure, adverse effects, immune checkpoint inhibitors

## Abstract

We report a case of a 58-year-old woman with secondary adrenocortical insufficiency due to adrenocorticotropic hormone (ACTH) deficiency after pembrolizumab treatment that required differentiation from low cardiac output syndrome. The patient had chronic heart failure due to radiation cardiomyopathy and underwent implantation of cardiac resynchronization therapy defibrillator (CRT-D). One year ago, she was diagnosed with squamous cell lung cancer and started combination therapy with carboplatin, paclitaxel, and pembrolizumab. She was hospitalized for anorexia, nausea, and hypotension. A diagnosis of secondary hypoadrenocorticism due to isolated ACTH deficiency was made, and from the course of the disease, it was diagnosed as a side effect of immune checkpoint inhibitors (ICIs). As the indications for ICIs continue to expand, it is necessary to understand the screening and management of their side effects.

## Introduction

Immune checkpoint inhibitors (ICIs) are currently used in many types of cancer, and their indications are expected to further expand in the future. Immune-related adverse events (irAEs) are specific to ICIs [1]. Patients with cancer often complain of general malaise, shortness of breath, edema, and other symptoms due to the progression of the disease or cancer treatment, and it is often difficult to identify the cause from the symptoms alone. We report a case of secondary adrenocortical insufficiency developed due to adrenocorticotropic hormone (ACTH) deficiency after pembrolizumab treatment that required differentiation from low cardiac output syndrome.

## Case presentation

The patient was a 58-year-old woman. Thirteen years ago, she had undergone surgery and radiation therapy for a mediastinal tumor. The left lung field was irradiated with approximately 53 Gy. She had a family history of cancer and heart disease; however, the details were unknown. She did not smoke or drink alcohol and was independent in terms of activities of daily living until one year ago when she was treated with anticancer drugs. Seven years ago, she was hospitalized for the first time due to heart failure and transthoracic echocardiography (TTE) revealed a left ventricular ejection fraction (LVEF) of 30%. She was diagnosed with radiation cardiomyopathy after a thorough examination. She was repeatedly hospitalized for heart failure, and three years ago, she had undergone implantation of a cardiac resynchronization therapy defibrillator (CRT-D) due to the appearance of a complete atrioventricular block and non-sustained ventricular tachycardia. One year ago, she was diagnosed with squamous cell lung cancer and started combination therapy with carboplatin, paclitaxel, and pembrolizumab. After four courses, she developed nausea and worsening shortness of breath, and her performance status dropped; therefore, she was followed up without maintenance therapy. Two months prior, she was hospitalized for nausea and worsening shortness of breath and was discharged after adjusting for diuretics and CRT-D settings. However, nausea soon flared up again, and diuretics were uptitrated on an outpatient basis, but she was admitted to our hospital with symptomatic hypotension (systolic blood pressure of 60 mmHg). The chief complaints were anorexia, nausea, and hypotension. She was taking tolvaptan (15 mg), spironolactone (50 mg), and azosemide (15 mg) as diuretics, enalapril (1.25 mg) and bisoprolol (5 mg) as heart failure therapy, and edoxaban (30 mg) to prevent stroke due to paroxysmal atrial fibrillation. However, when she visited the hospital, she was unsteady and had difficulty going to the toilet.

**Figure 1:**
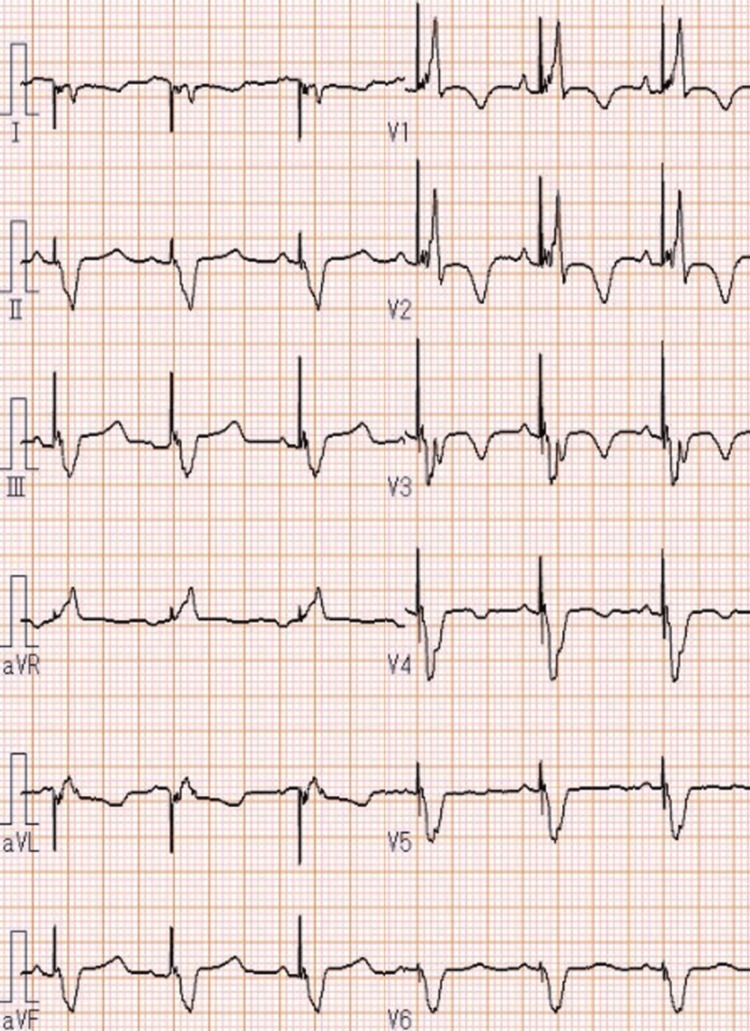
A 12-lead ECG showed a heart rate of 84 beats/minute in sinus rhythm and a biventricular pacing waveform.

At the time of admission, her body mass index was 19.1 kg/m^2^. Her blood pressure was 60/47 mmHg, pulse rate was 91/minute, and oxygen saturation was 97% on room air. A grade 4/6 systolic murmur was heard at the apex of the heart as the strongest point. Her peripheral extremities were cold and dry. A 12-lead electrocardiogram (ECG) showed a heart rate of 84 beats/minute in sinus rhythm and a biventricular pacing waveform that had not changed significantly since the previous discharge (Figure 1).

**Figure 2:**
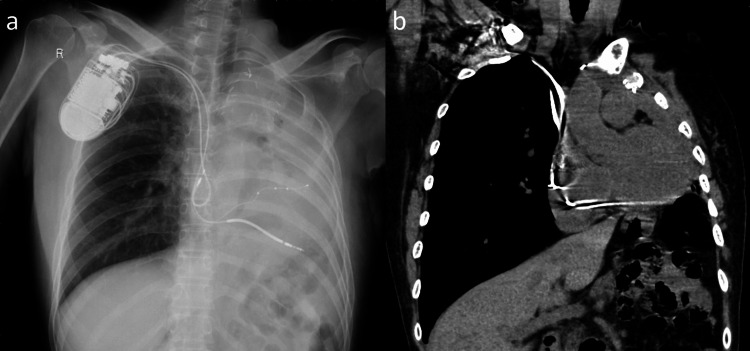
A chest radiograph (a) and a computed tomography (b) showed negligible permeability in the left lung field owing to postoperative effects, and a cardiac resynchronization therapy defibrillator was implanted in the right anterior thoracic region. There was no pleural effusion.

A chest radiograph and a computed tomography showed negligible permeability in the left lung field owing to postoperative effects, and the CRT-D device in the right anterior thoracic region. There was no pleural effusion or significant change from the previous discharge (Figure 2).

TTE revealed the left ventricular (LV) end-diastolic and end-systolic dimensions were 49 and 41 mm, respectively. The LVEF was 39%, and moderate aortic and mitral regurgitation were detected. The size of the inferior vena cava was 14 mm during expiration and the estimated pulmonary arterial pressure was 39 mmHg. There was no significant change from the echocardiographic study at the time of the previous discharge.

On admission, laboratory investigation (Table 1) revealed a brain natriuretic peptide level of 160.9 pg/mL, which was low (she had been at 300-500 pg/mL in the outpatient setting).

**Table 1:** Laboratory data on admission.

Test	Value	Unit	Normal range
White blood cell	5540	/μL	3300-9600
Neutrophil	62.3	%	40.0-75.0
Eosinophil	6.0	%	0.0-8.5
Lymphocyte	21.5	%	16.5-49.5
Red blood cell	474	10^4^/μL	386-492
Hemoglobin	11.9	g/dL	11.6-14.8
Hematocrit	37.4	%	35.1-44.4
Platelet	26.5	10^4^/μL	15.8-34.8
Total protein	6.3	g/dL	6.6-8.1
Albumin	3.5	g/dL	4.1-5.1
Total bilirubin	0.5	mg/dL	0.4-1.5
Aspartate aminotransferase	26	U/L	13-30
Alanine aminotransferase	11	U/L	7-23
Lactate dehydrogenase	226	U/L	124-222
γ-glutamyl transpeptidase	12	U/L	9-32
Blood urea nitrogen	7.8	mg/dL	8.0-20.0
Creatine	0.9	mg/dL	0.46-0.79
Uric acid	5.1	mg/dL	2.6-5.5
Sodium	137	mmol/L	138-145
Potassium	4.3	mmol/L	3.6-4.8
Chloride	103	mmol/L	101-108
Creatine kinase	41	U/L	41-153
Brain natriuretic peptide	160.9	pg/mL	<20.0
C-reactive protein	0.36	mg/dL	<0.14
Total cholesterol	168	mg/dL	142-248
Triglyceride	89	mg/dL	30-149
High-density lipoprotein cholesterol	40	mg/dL	40-103
Low-density lipoprotein cholesterol	104	mg/dL	65-139
Fasting blood sugar	89	mg/dL	73-109
Hemoglobin A1c	6.2	%	4.9-6.0
Troponin I	0.01	ng/mL	<0.04

Endocrine biochemical findings (Table 2) revealed 1.9 pg/mL (2.1-3.8) free triiodothyronine, 8.0 pg/mL (7.7-63.3) ACTH, and 0.8 μg/dL (2-18) cortisol.

**Table 2:** Endocrinological laboratory data.

Test	Value	Unit	Normal range
Thyroid-stimulating hormone	0.531	μU/mL	0.50-3.00
Free triiodothyronine	1.9	pg/mL	2.1-3.8
Free thyroxine	1.2	ng/dL	0.8-1.5
Adrenocorticotropic hormone	8.0	pg/mL	7.7-63.1
Cortisol	0.8	μg/dL	2-18
Growth hormone	0.5	ng/mL	≦0.13
Prolactin	24.5	ng/mL	3.6-12.8
Luteinizing hormone	43.1	mIU/mL	1.2-7.1
Follicle-stimulating hormone	84.5	mIU/mL	2.0-8.3

**Figure 3:**
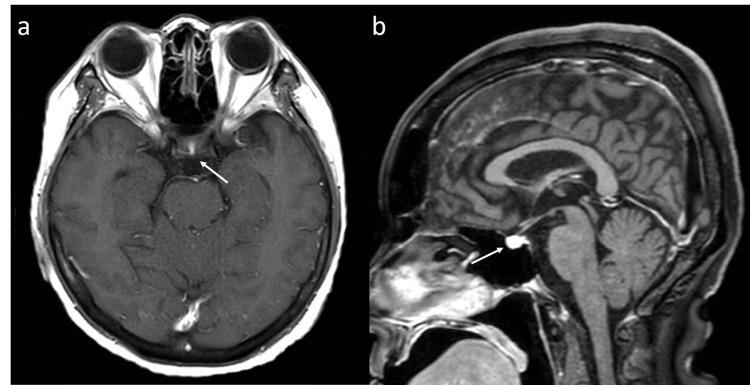
Magnetic resonance imaging of the head revealed no obvious abnormal findings in the pituitary gland (arrows) (a, sagittal plane; b, horizontal plane). Intracranial metastatic lesions were not observed.

After consulting the division of endocrinology and metabolism, a clinical diagnosis of adrenal insufficiency was made, and treatment with 100 mg hydrocortisone infusion was started on the same day. Her symptoms improved quickly, and she was able to eat. Her systolic blood pressure increased to 90 mmHg. After her condition stabilized, the hydrocortisone infusion was switched to oral hydrocortisone (15 mg/day). The results of the corticotropin-releasing hormone (CRH) test showed that ACTH levels were 3.2/6.4/10.1/7.3/6.8/5.1 pg/mL at pre/15/30/60/90/120 minutes. Tests for thyrotropin-releasing hormone, luteinizing hormone-releasing hormone, and growth hormone-releasing peptide 2 were also performed; however, no significant findings were observed. The peak ACTH level was more than twice the basal level, but the patient was hyporesponsive at 10.1 pg/mL. Magnetic resonance imaging of the head revealed no obvious abnormal findings in the pituitary gland (Figure 3).

Intracranial metastatic lesions were not detected. Based on these results, a diagnosis of secondary hypoadrenocorticism due to isolated ACTH deficiency was made, and from the course of the disease, it was diagnosed as a side effect of ICI. The patient continued to perform well and was discharged from the hospital with continued oral hydrocortisone administration.

## Discussion

In cancer patients with chronic heart failure, it is often difficult to differentiate symptoms due to cancer from those due to cancer treatment and heart failure. Careful medical differential diagnosis is important. It has been suggested that ICI-induced adverse events may have a slower onset and longer duration than chemotherapy-induced adverse events. It should be noted that in some cases, irAEs may develop months or years after treatment discontinuation [2]. Although the incidence of ICI-associated irAEs is not high, safety management is important because the sites, types, and timing of their onset are diverse and difficult to predict [3]. ICI-related myocarditis can occur with acute heart failure, pulmonary edema, and, in severe cases, cardiogenic shock, multiorgan failure, and ventricular arrhythmias, and can lead to death [4,5]. The incidence of cardiac failure was 0.4% with pembrolizumab monotherapy [4]. In the case of cardiogenic shock, inotropic support and advanced mechanical support are needed.

Reports classify ICI-induced pituitary dysfunction into two types: hypopituitarism (pituitary enlargement and decreased secretion of multiple anterior pituitary hormones) and isolated ACTH deficiency (no pituitary enlargement, only decreased secretion of ACTH) [6]. The anti-PD-1 antibody used in this case has been reported only for isolated ACTH deficiency, with a frequency of 6% [7]. In addition, a significant increase in overall survival was observed in patients with pituitary-irAE compared to those without in both malignant melanoma and non-small cell lung carcinoma patients, suggesting that proper diagnosis and replacement therapy for pituitary-irAE may improve activities of daily living and quality of life [8]. In this case, the diagnosis of isolated ACTH deficiency was made based on the lack of ACTH secretory response to CRH stimulation and preserved secretion of growth hormone (GH), thyroid-stimulating hormone, luteinizing hormone (LH), follicle-stimulating hormone, and prolactin. Although the CRH study was performed after steroid replacement therapy, we do not believe that steroid replacement therapy has any effect on the suppression of the ACTH secretion in anterior pituitary cells. It is presumed that the specific autoimmune mechanism of pembrolizumab against ACTH-producing cells causes isolated ACTH deficiency, which leads to the development of secondary adrenocortical insufficiency. It is desirable to perform available tests such as blood tests for early detection, and we believe that it is necessary to establish a routine with some predefined items in the future.

## Conclusions

We encountered a case of secondary adrenocortical insufficiency developed due to ACTH deficiency after pembrolizumab treatment that was difficult to differentiate from low cardiac output syndrome. Treatment with hydrocortisone improved the patient’s symptoms and hypotension quickly. As the indications for ICI continue to expand, it is necessary to understand the screening and management of their side effects.
